# Bistable Soft Shells for Programmable Mechanical Logic

**DOI:** 10.1002/advs.202412372

**Published:** 2024-12-06

**Authors:** Nan Yang, Yuming Lan, Miao Zhao, Xiaofei Shi, Kunpeng Huang, Zhongfa Mao, Damiano Padovani

**Affiliations:** ^1^ Intelligent Manufacturing Key Laboratory of the Ministry of Education College of Engineering Shantou University Shantou 515063 China; ^2^ School of Mechanical and Electrical Engineering University of Electronic Science and Technology of China Sichuan 611731 China; ^3^ Department of Mechanical Engineering (Robotics) Guangdong Technion‐Israel Institute of Technology Shantou 515063 China

**Keywords:** interchangeable surfaces, mechanical computing, negative stiffness, programmable devices, Soft shells

## Abstract

Mechanical computing promises to integrate semiconductor‐based digital logic in several applications, but it needs straightforward programmable devices for changing computing rules in situ. A methodology based on strain‐governed, bistable soft shells that process digital information by interchanging their internal/external surfaces is proposed. This bistable behavior, explained via model‐based design, safeguards robustness by working only once for each input pulse. Thus, these shells are leveraged to create a buffer and a NOT gate that lead to six fundamental gates (AND, OR, NAND, NOR, XOR, and XNOR). All these functions are integrated into a unique programmable device, making mechanically integrated circuits more adaptable with rule‐changeable logic operations. This design ensures continuous processes and general applicability to multiple types of signals (a pressurized fluid can replace mechanical driving signals is shown). It also empowers more complex logic functions suitable for expanded applications, such as the half and full adders is addressed.

## Graphical abstract

Traditional computing methods, such as semiconductor‐based digital logic, suffer from failures in extreme conditions. A general approach for forming electronic‐free computers is proposed to overcome this barrier by leveraging bistable soft shells. A programmable device that achieves many Boolean logic rules in situ is developed. This approach eliminates the individual design of each logic gate and ensures continuous operations.

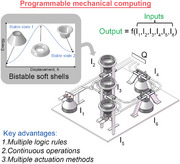

## 1 Introduction

Even if electronic (semiconductor‐based) computation and information processing represent today's standard, mechanical computing systems are flourishing again, presenting as a potential supplement. They can interact with their environment by conveniently merging concepts from information science, robotics, and materials science.^[^ ^1^ ^]^ Mechanical computing relies nowadays on modern solutions based on soft metamaterials architected in space and time.^[^ ^2^ ^]^ Their inherent advantages are dictated by unusual properties and new functionalities, such as attaining different mechanical characteristics,^[^ ^3^, ^4^, ^5^, ^6^ ^]^ or performing programmable structural transformations,^[^ ^7^, ^8^, ^9^, ^10^, ^11^ ^]^ Information processing takes place mechanically by leveraging the motion or deformation of suitable devices; these features enable signal transmission,^[^ ^12^, ^13^, ^14^, ^15^ ^]^ information storage,^[^ ^16^, ^17^, ^18^, ^19^, ^20^ ^]^ and logic operations,^[^ ^21^, ^22^, ^23^, ^24^, ^25^, ^26^, ^27^, ^28^ ^]^ Focusing on the latter area, executing digital logic based on different mechanisms has been proposed, such as using DNA molecules,^[^ ^29^ ^]^ micro‐electro‐mechanical systems,^[^ ^30^ ^]^ optical devices,^[^ ^31^, ^32^, ^33^, ^34^ ^]^ artificial neurons and synapses,^[^ ^35^ ^]^ thermal switches,^[^ ^36^ ^]^ and fluidic elements,^[^ ^37^, ^38^, ^39^, ^40^ ^]^ However, using mechanical integrated systems makes it easier to perform binary Boolean operations as responses to environmental stimuli.^[^ ^1^ ^]^

Mechanical logic gates are the building blocks of mechanical computing and contain two discrete and stable configurations representing the “0” and “1” binary states. These gates can be implemented as rotary joints connected by links,^[^ ^41^ ^]^ flexure mechanisms,^[^ ^42^ ^]^ waterbomb origami,^[^ ^43^ ^]^ or strain‐governed soft modules.^[^ ^44^ ^]^ Ensuring general applicability and enhanced computation capabilities requires programmable logic gates. An initial attempt delivered a device incapable of continuous operations.^[^ ^13^ ^]^ A reconfigurable mechanism implemented nine logic operations by modifying its stability behavior^[^ ^45^ ^]^ but with a delay in propagating the input.^[^ ^12^ ^]^ A 3‐D anisotropic unit cell performed AND/OR logic based on its buckling response to actuation^[^ ^46^ ^]^; the direction of actuation plays a crucial role in the logic sequence, as opposed to previous metamaterials actuated in a single plane,^[^ ^12^, ^13^, ^27^, ^42^, ^45^ ^]^ Then, a reprogrammable metamaterial achieved logic operations by relating its local deformation with information processing,^[^ ^47^ ^]^ but requiring electrical excitation to drive electromagnets. Modulating the alternate current's driving frequency is the case for a reprogrammable microelectromechanical resonator, which performs all the fundamental 2‐bit logic operations.^[^ ^48^ ^]^ Bistability can also be obtained from intrinsically monostable structures via a controlled magnetic field,^[^ ^49^ ^]^ even if with limited reconfigurations. A mechanical binary neural network implemented the sixteen logic gates feasible with two inputs and one output,^[^ ^50^ ^]^ despite its bulky design and complex functioning prevent an easy implementation. Therefore, rule‐changeable logic gates characterized by multiple rules and operating continuously by performing operations in series (both identical and different logic rules) represent a gap in technical literature.

We propose, for this reason, a methodology for mechanical computation based on bistable soft shells that can (1) handle multiple logic rules in situ in a streamlined and programmable configuration (i.e., go beyond the basic AND gate and OR gate by easily switching between them all), (2) ensure continuous operations (i.e., remove the need for resets before starting a new computation), and (3) permit mechanical excitation alone but also allow for general applicability (i.e., use force, pressure, light, or electricity as inputs and outputs). After introducing the soft shells that are the pivotal element of this study, we discuss their arrangement to build fundamental and complex logic gates. We then present the experimental validation of our programmable device before concluding the discussion with final remarks. Furthermore, we complement this article by exhaustively illustrating our methods and additional results in the Supporting Information.

## 2 Results and Discussion

We start by introducing the proposed design of the soft shells and evaluating their mechanical properties. We then present our fundamental logic gates and complex computing devices.

### 2.1 Functional Design of the Soft Shells

**Figure 1:**
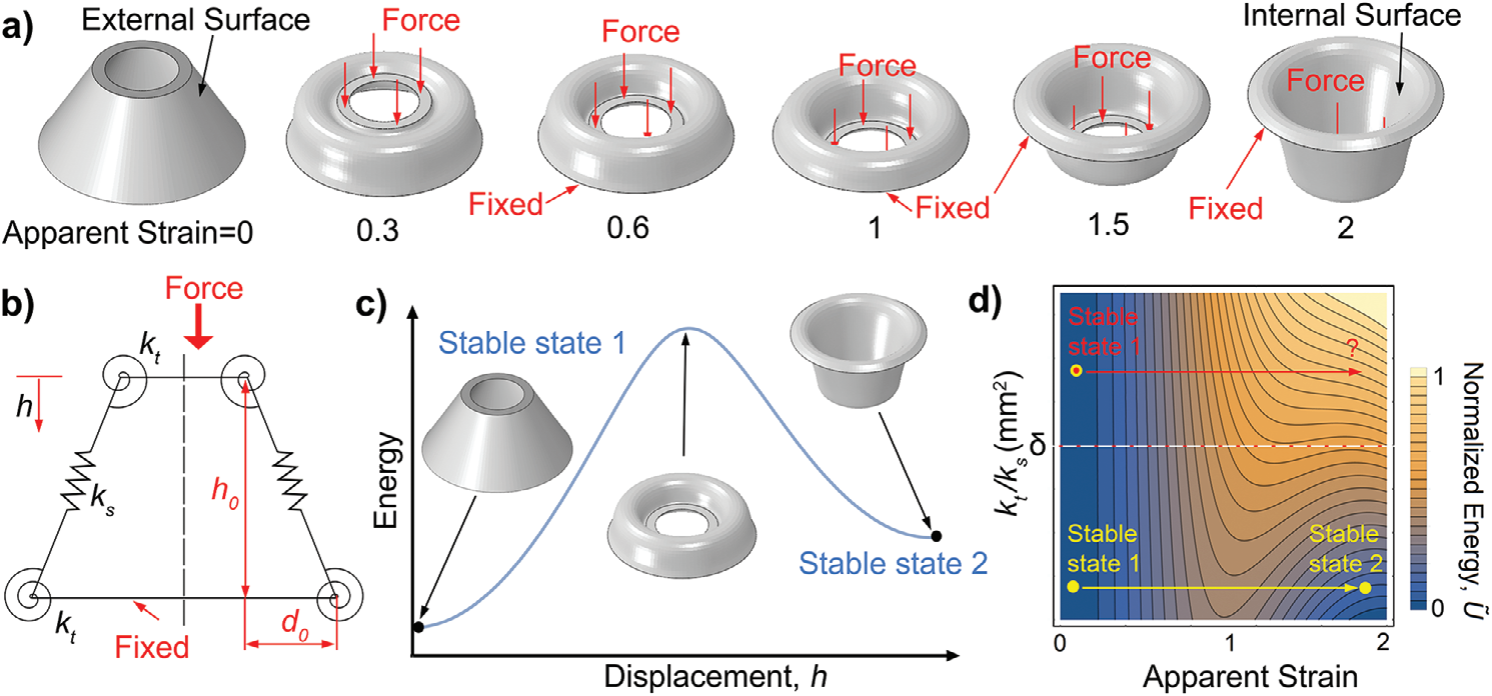
Overview of the bistable soft shells proposed in this research. a) Shell design and stages of the deformation process when an external force is applied on the shell's top edge while the bottom edge is fixed. b) A simplified representation of the shell suitable for an analytical model with equivalent torsion and linear springs (this model provides an intuitive understanding of the shell behavior). c) Qualitative trend of the elastic potential energy‐displacement curve of a bistable shell resulting from the analytical model depicted in panel (b). d) Contour plot of the normalized shell's potential energy as a function of both the spring stiffnesses’ ratio *k_t_*/*k_s_* and the shell's apparent strain (the plot indicates a bistable design is possible by selecting a proper ratio *k_t_*/*k_s_*, where δ ≈ 80 mm^2^).

We design a shell with the shape of a circular truncated cone, which has a bistable configuration leading to interchangeable surfaces, as shown in **Figure** **1**a. When a sufficient compressive force is applied on the top part of the shell while the bottom part is fixed, the external surface of the shell (apparent strain *s*  =  0) becomes its internal surface with the apparent strain increasing to its maximum value (*s*  =  2). We name it “apparent strain” to avoid confusion with the rigorous definition of strain used in the shallow shell theory.^[^ ^51^, ^52^ ^]^ Here, it is defined by *s*  =  *h*/*h*_0_ and takes values [0, 2], where *h* and *h*_0_ denote the shell's compressive displacement and initial height, respectively. To qualitatively understand the mechanical behavior of the shell, we use an analytic model with four torsion and two linear springs (Figure 1b) to define the total elastic potential energy *U* (see SM for more details). We can write it as:

(1) $$U/k_{s}={\left(l-l_{0}\right)}^{2}+2\frac{k_{t}}{k_{s}}{\left(\theta -\theta_{0}\right)}^{2}$$

so that the normalized potential energy is defined as $\tilde{U}=\frac{\frac{U}{k_{s}}}{max(\frac{U}{k_{s}})}\;\leq 1$, where *k_t_* and *k_s_* are the stiffnesses of the torsion and linear springs, respectively, *l* − *l*_0_ denotes the length difference between the linear spring and its original state, and θ − θ_0_ indicates the angle difference between the torsion spring and its original state. If the ratio *k_t_*/*k_s_* is appropriately chosen, the shell has two stable states (the dots in Figure 1c denotes local minimum values of energy). As shown in the dimensionless contour plot involving the ratio *k_t_*/*k_s_* and the normalized potential energy $\tilde{U}$, there are two stable states when *k_t_*/*k_s_* < δ (they are located below the dashed line in Figure 1d, where δ ≈ 80 mm^2^) so that the shell is bistable; otherwise, there is only one stable state situated above the dashed line when *k_t_*/*k_s_* > δ, so the shell is monostable. The proposed bistable shell structure has two benefits in designing mechanical logic gates. Each shell state can be easily maintained without continuously applying an external input, thus simplifying operations, and robust functioning is enabled by changing the shell state only once for each (impulsive) input signal.

### 2.2 Mechanical Properties of the Soft Shells

**Figure 2:**
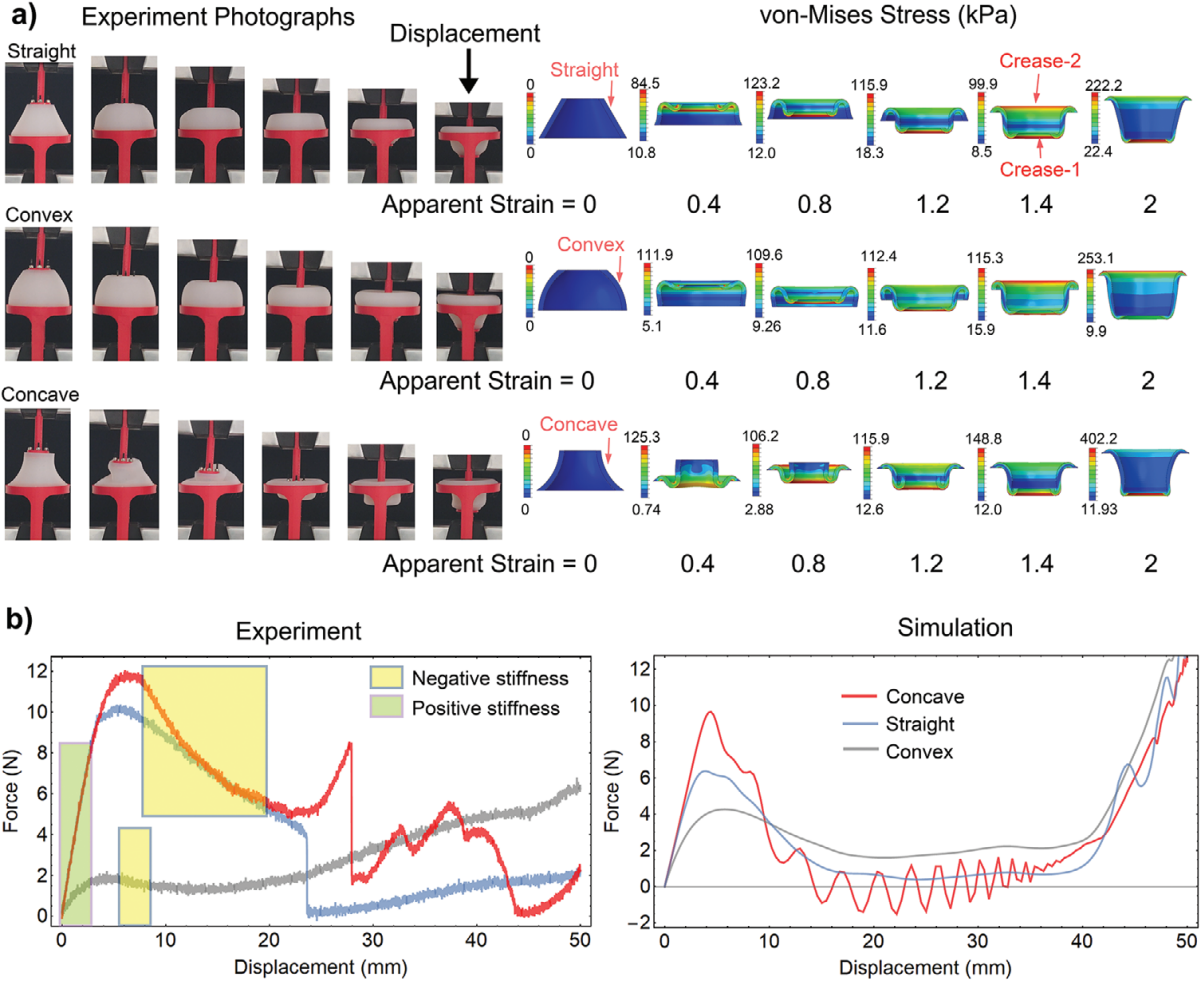
Testing three types of soft shells (i.e., straight, convex, and concave units) specifically prototyped for quasi‐static mechanical experiments. a) Experimental photographs and numerical simulations of the shells during different deformations ranging from no deformation on the left‐hand side (apparent strain *s*  =  0) to maximum deformation on the right‐hand side (*s*  =  2). b) Experimental and simulated trends of the shells’ force‐displacement curves during the entire deformation process (very low values of the applied force for extreme deformations indicate a bistable behavior of the soft shell).

To find the optimal shape, we designed three versions of the soft shells with the same height: straight, convex, and concave, as labeled in **Figure** **2**a. We targeted table‐scale dimensions (the exact values can be seen in Figure S1, Supporting Information) to ensure easy prototyping of the soft shells since this process is performed manually (Figure S2, Supporting Information). However, these components can be significantly downscaled to enhance compactness, especially if a silicone printer is available. We connected the three types of soft shells to the upper and lower holders and compressed them with a universal testing machine to explore their mechanical properties (these experiments confirmed the intuitive analysis built on the theoretical model we presented above). In Figure 2a, we report the experimental and numerical conditions representative of six states with different apparent strains. Each shell type deals with compressive forces at the beginning of the test (0 ≤ s < 1), but handles tensile forces later (s > 1) with the consequent interchange of the internal/external surfaces. To clearly show the stress distribution simulated in the component with a finite element analysis, we present cross‐sectional views of the shells in Figure 2a. We can notice the stress is located in specific regions of the shell based on the apparent strain; for instance, two creases are highlighted in the figure when s  =  1.4. In terms of overall behavior, we addressed it by calculating the shell's equivalent stiffness based on the measured force that produces the deformation. Both straight and concave units show high values of negative stiffness (see the highlighted areas in Figure 2b); the slope of their experimental force‐displacement curves resulted equal to −0.4 ± 0.02 and − 0.61 ± 0.003 N mm^−1^, respectively. This aspect is a sign of bistability because the force value tends to approach zero, which corresponds to minimum potential energy, for extreme deformations at both ends of the spectrum. Conversely, the convex shell has a reduced negative stiffness equal to −0.07 ± 0.005 N mm^−1^, which implies monostability because the applied force does not drop for high deformations (i.e., the shell retains significant potential energy). Focusing on the elastic region (refer to the shaded area for small deformations in Figure 2b), the straight and concave shells show similar numbers of positive stiffness (2.94 ± 0.01 and 2.86 ± 0.07 N mm^−1^, respectively). They are much higher than that of the convex shell, namely 0.52 ± 0.01 N mm^−1^, confirming that straight and concave units respond similarly to deformation. In Figure 2b, the results of the experiments and simulations match well in the low‐displacement region both qualitatively and quantitatively but diverge for very high deformations, especially for the concave and straight shell design. This characteristic suggests that such a finite element model is proper for simulations with small deformations. Nevertheless, developing a more accurate model goes beyond the scope of this paper since the experimental trends are supported well enough to make an educated decision about the shell shape. Due to the higher negative stiffness, which favors the bistable behavior, and the ease of fabrication, we chose the shell design based on a straight cone profile to create the soft computing units for the following experiments. We selected a cone angle of 30 degrees (it requires an intermediate value of the actuation force, as reported in Figure S3, Supporting Information) and ensured the resulting shell design is robust enough for repeated operations (Figure S4, Supporting Information).

### 2.3 Fundamental Logic Elements

**Figure 3:**
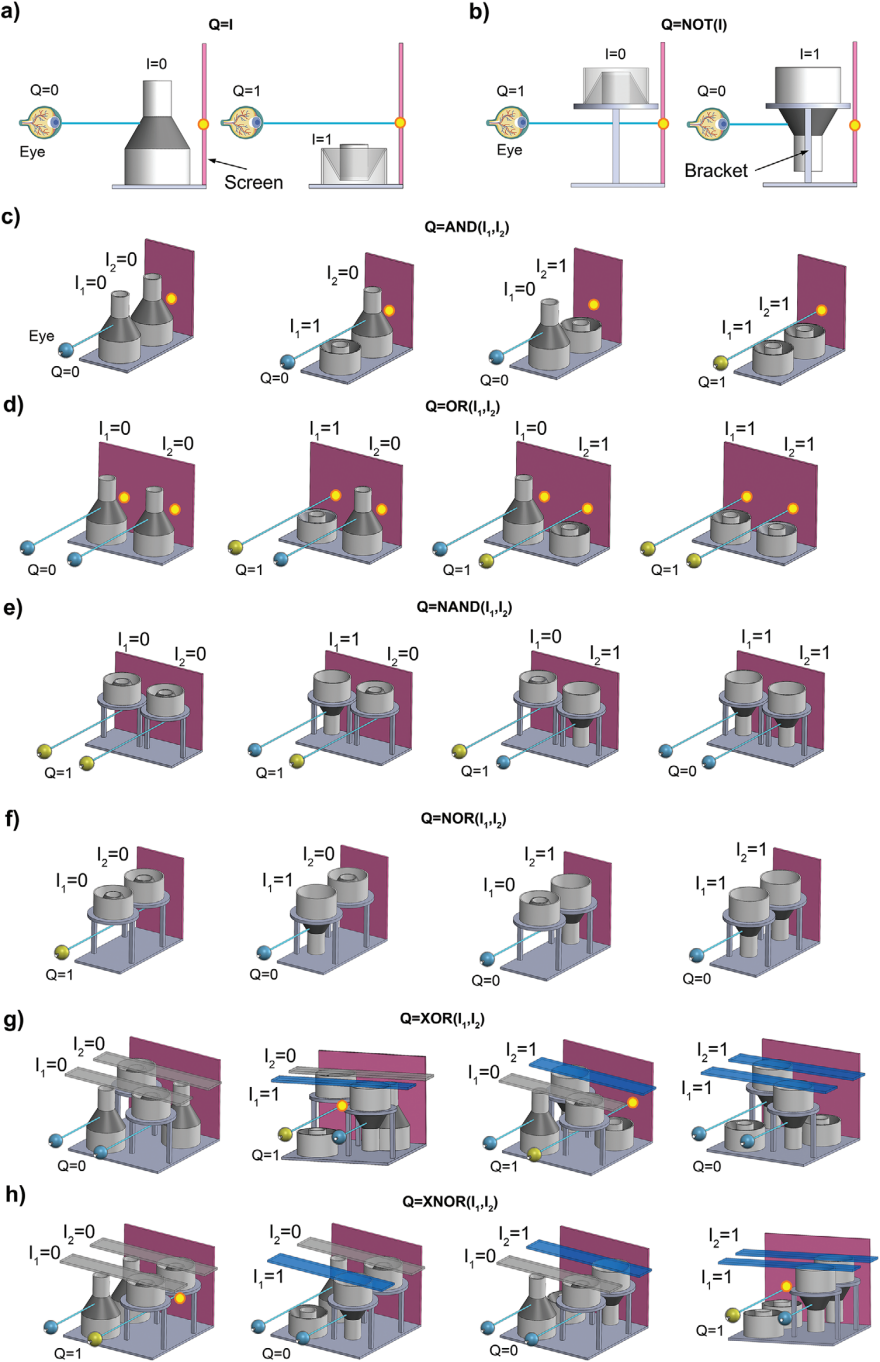
System diagrams of the six proposed fundamental logic elements characterized by bistable behavior: a) Buffer unit, b) NOT gate, c) AND gate, d) OR gate, e) NAND gate, f) NOR gate, g) XOR gate, and h) XNOR gate. The soft units’ inputs *I* and outputs *Q* are specified (*I* corresponds to the triggering force applied/unapplied on the shell while *Q* refers to the enabled/disabled target visualization highlighted by yellow dots on the purple device's screen). The user's point of view is given (the operator eye), and the interrupted/uninterrupted lines of sight are also represented (segments in blue). The XOR and XNOR gates in panels (g) and (h) are based on two buffers and two NOT gates, so their two inputs are indicated by equivalent strips that trigger two basic units simultaneously. Potential implementations of all these elements are shown in the SM (see Figures S5–S7, Supporting Information).

We initially applied the abovementioned soft shells to propose two foundational logic elements, namely a buffer unit and a NOT gate. We augmented the shell design by adding two cylindrical sections with reduced thickness below and above the conical section (see Figure S2d, Supporting Information); this approach facilitates the installation of the shells and the selection of the stable equilibrium states. These soft shells can perform basic logic and arithmetic operations purely mechanically without other technologies (e.g., electric or magnetic actuating elements). To explain the proposed functioning of our set‐ups, let's suppose the user is monitoring a target on the device screen. We define the device output as equal to the logic state *Q*  =  0 if the operator eye cannot see the target. Otherwise, the output turns to the logic state *Q*  =  1 when the user visualizes the target. Starting with the **buffer unit** shown in **Figure** **3**a, the system output, or buffer state, is *Q*  =  0 if the user view is blocked by the bistable unit, that results deactivated (i.e., the system input is *I*  =  0 because the upper cylindrical section of the soft shell, simply the trigger, is not pressed down). When the unit is activated (i.e., the input is *I*  =  1 since the trigger is pressed down), the vision of the target takes place, and the unit outputs *Q*  =  1. On the other hand, a **NOT gate** can be realized by inverting the positioning of a buffer unit and installing it on a support bracket (Figure 3b). We describe its functioning as *Q*  =  NOT(*I*) that is the opposite of the buffer unit because we consider the shell not to be triggered when it is in its squeezed state; thus, the gate can perform the proper function (i.e., input *I*  =  0 and output *Q*  =  1).

We can now realize multiple computing rules by joining buffer units and NOT gates; in detail, we develop six fundamental logic elements as follows. When two buffers are placed in series (Figure 3c), we obtain an **AND gate** with an output response *Q*  =  AND(*I*_1_,*I*_2_). The user eye can only see the target (*Q*  =  1) if both units are triggered (*I*_1_ = *I*_2_  =  1), whereas the target view is blocked in all other cases (*Q*  =  0). Then, we create an **OR gate** outputting *Q*  =  OR(*I*_1_,*I*_2_) by arranging two buffers in parallel (Figure 3d). The user visualizes the target (*Q*  =  1) when, at least, one unit is triggered (*I*_1_ =  1, *I*_2_ =  1, or  *I*_1_ =  *I*_2_ =  1). The target view is only blocked (*Q*  =  0) when both inputs are null ( *I*_1_ =  *I*_2_ =  0).

Moreover, we form a **NAND gate** leading to *Q*  =  NAND(*I*_1_,*I*_2_) when placing two NOT gates in parallel (Figure 3e). The user does not see the target (*Q*  =  0) if and only if both units are triggered ( *I*_1_ =  *I*_2_ =  1). Conversely, the target is visible (*Q*  =  1) when at least one unit is deactivated (*I*_1_ =  0, *I*_2_ =  0, or  *I*_1_ =  *I*_2_ =  0). Arranging two NOT units in series leads to a **NOR gate** outputting *Q*  =  NOR(*I*_1_,*I*_2_), as depicted in Figure 3f. The user visualizes the target (*Q*  =  1) only when both units are deactivated ( *I*_1_ =  *I*_2_ =  0). The target view is, therefore, blocked (*Q*  =  0) if, at least, one unit is triggered (*I*_1_ =  1, *I*_2_ =  1, or  *I*_1_ =  *I*_2_ =  1).

Furthermore, we can achieve additional logic functions by combining four basic units (buffers and NOT gates) and activating two of them simultaneously with a single input command. We realize an **XOR gate** with output *Q*  =  XOR(*I*_1_,*I*_2_) by arranging two buffers and two NOT units, as shown in Figure 3g (a buffer and a NOT gate are positioned in series along the lines of sight). The entire device outputs *Q*  =  0 when both targets are not visible because the two inputs are equal (*I*_1_ = *I*_2_). The figure indicates these inputs with transparent strips above the shells, where *I_i_* =  1 means the buffer and NOT gate are pressed down. Conversely, the XOR gate outputs *Q*  =  1 (at least one target is visible) when the two inputs differ (*I*_1_ ≠ *I*_2_). We can also install the same basic units according to the pattern reported in Figure 3h (two buffers are placed in series along one line of sight, while two NOT gates are put in series along the other line of sight). This approach forms an **XNOR gate** with a response *Q*  =  XNOR(*I*_1_, *I*_2_). The device outputs *Q*  =  1 (at least one target is visible) when both inputs are equal (*I*_1_ = *I*_2_); if the two inputs are different (*I*_1_ ≠ *I*_2_), the output becomes *Q*  =  0 since both targets are not visible.

As a final remark, these fundamental logic gates serve as the building blocks of more complex devices that do not represent the object of the present study. We can leverage them to design, for instance, a **half adder** or a **full adder** (Figure S8, Supporting Information); these results support the adaptability of the suggested approach to set up mechanically integrated circuits for computation beyond simple logic.

### 2.4 Experimental Validation

**Figure 4:**
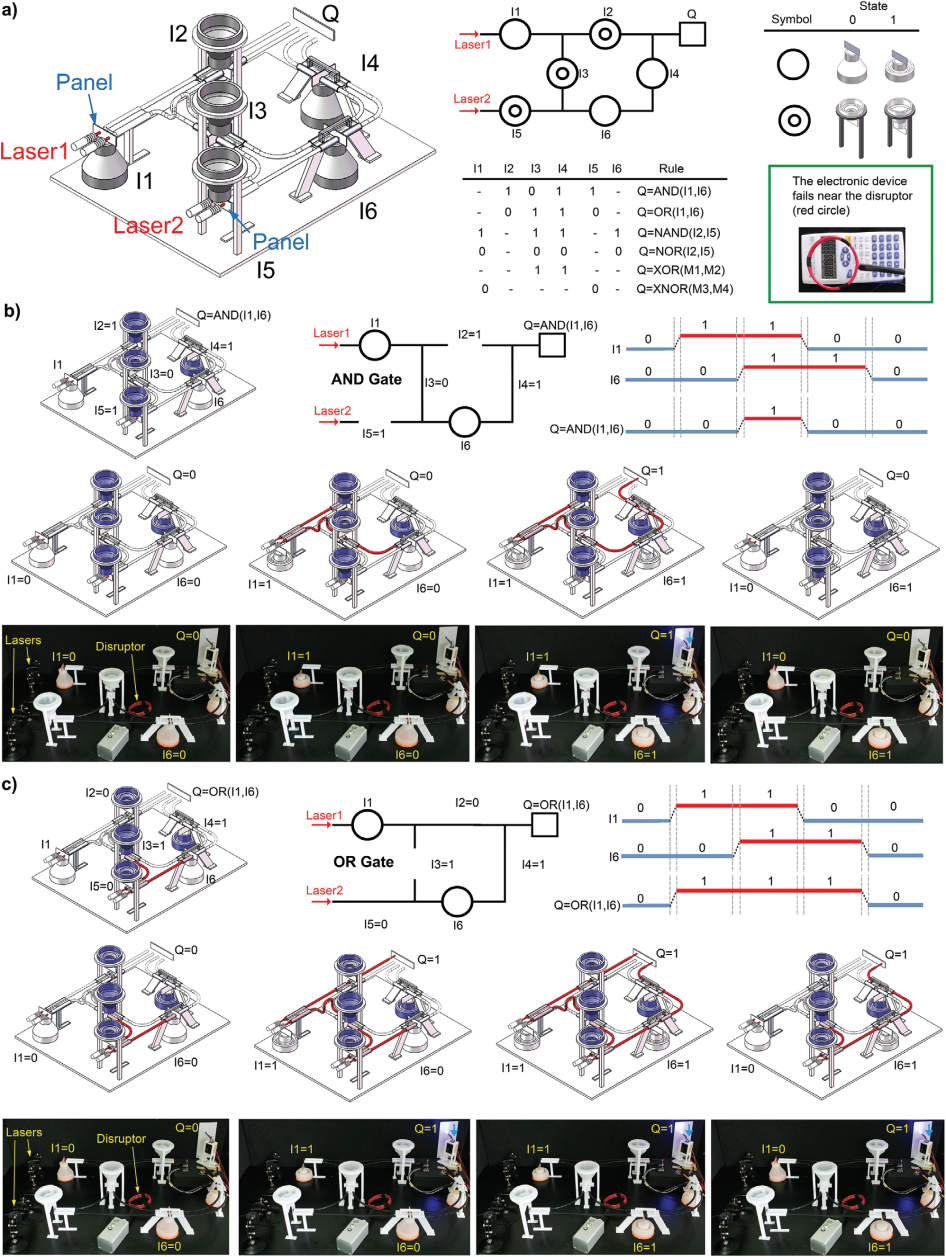
The proposed rule‐changeable device performing all the essential logic gates used to implement Boolean functions in computing systems. a) Overview of the device, its equivalent diagram (the system is represented at rest because the soft shells are not triggered), a table elucidating the meaning of the symbols, its truth table with the corresponding settings, and a proof of the failure of an electronic device when placed near a disruptor constituted by the red circle (lower right inset). b) Example of the AND gate (the soft shells highlighted in blue form the AND gate, while the remaining two shells define the gate inputs). c) Example of the OR gate. Panels (b,c) report the overview of different scenarios, the equivalent diagram, a chart of continuous operations of the inputs and outputs, and photographs of the four states during experimental validations (our system is tested under the action of the disruptor). The other logic gates achievable with this device (AND, NAND, NOR, XOR, and XNOR) are reported in the SM (see Figures S9 and S10 (Supporting Information) for the system models and experimental photographs, respectively). The laser sensors located at the optical fibers’ end and the corresponding relays used to visualize the output *Q* can be seen in Figure S11 (Supporting Information) (*Q*  =  1 when, at least, one sensor receives the laser beam). Such a system can be extended to construct a network of gates that realizes more combinational logics (Figure S12, Supporting Information illustrates an example).

We have designed a rule‐changeable system with six soft shells characterized by six independent inputs (*I*_*i*= 1, 2, 3, 4, 5, 6_). It realizes multiple functions by switching between the six logic gates depicted above quickly and in a straightforward manner (see panels (c–h) in Figure 3). **Figure** **4**a shows this rule‐changeable device relies on an automated optical system with two groups of laser emitters (four lasers in total) and one photoelectric converter (P/E, including a sensor and a relay JQC‐3FF‐S‐Z), where a network of optical fibers carries the laser beams. The bistable shells allow/block the transmission of the laser beams, depending on their input value (1 or 0), via an attached panel (see the state diagram in Figure 4a). The reason for using small panels to interact with laser beams is to shorten the distance between adjacent optical fibers, making the device more compact and effective. If the laser beam passes and hits the P/E, then the output *Q*  =  1 is displayed by an LED light. In particular, we use three buffers (they refer to the shells characterized by *I*_1_, *I*_4_ and *I*_6_ in Figure 4, such that *I*_*i*= 1, 4, 6_ = 0 leaves the corresponding shell extended to stop the laser beam) and three NOT gates (they refer to the shells characterized by *I*_2_, *I*_3_ and *I*_5_ in Figure 4, such that *I*_*i*= 2, 3, 5_ =  0 makes the corresponding shell retracted to let the laser beam pass). The truth table shows the required device settings to implement the targeted rule. For example, we obtain an AND gate by setting *I*_2_ = *I*_4_  = *I*_5_  =  1 and *I*_3_ =  0 (these shells, namely the “setting units”, are marked in blue in Figure 4b), while the inputs *I*_1_ and *I*_6_ perform the gate logic (they refer to the “operational units”). The gate settings and inputs are hand‐driven, as shown in Video S1 (Supporting Information). The equivalent diagram in the figure represents the AND gate by replacing the setting inputs *I*_*i*  =  2, 3, 4, 5_ with continuous/interrupted connections between elements to clearly highlight the gate inputs *I*_1_ and *I*_6_. This functioning can also be represented by a chart of continuous operations, where the digital values describe the relations between the inputs *I*_1_, *I*_6_ and the output *Q*. We also detail another example in this section to stress the rule‐changeable capability of this device, while the SM reports a complete overview of all the possible scenarios including the NAND, NOR, XOR, and XNOR gates. Thus, we enable an OR gate by setting *I*_2_ = *I*_5_  =  0 and *I*_3_ = *I*_4_  =  1 (Figure 4c). It is worth noticing the experimental photographs in Figure 4 illustrate the four states of each gate, where the output *Q*  =  1 means the blue light of the LED is ON and the moving element of the mechanical actuator pops down (see the blue arrows in the upper‐right corner of the photographs). The actuator is an optional component that is only used for visualizing the state of the input clearly; it adjusts its linear displacement depending on whether the laser beams hit the photoelectric converters, which generates corresponding commands on the relays used to operate the actuator (it draws energy from an external power supply according to the diagram in Figure S11, Supporting Information). Other components (e.g., LED) are also used to visualize the outputs clearly and can also be seen as optional components. These experiments demonstrate that our system can output electrical, optical, and force signals based on specific needs. It is also worth noticing that the device sustains stable signal propagations, leading to robust operations. Moreover, we placed our mechanical computing system near a disruptor (magnetic flux density ≤ 200 µ*T*) that generates electromagnetic disturbances (see the red circle in the photographs): our system can perform the operations as expected when the disruptor is working (see Video S2, Supporting Information), whereas an electronic device (TAKSUN, TS‐190) fails when the same electromagnetic disturbances are applied next to it (lower right inset in Figure 4a).

Considering an alternative activation of our programmable device shown in Figure 4a, we replaced the manual actuation of the soft shells with air‐driven actuation for enhanced applicability and remote operations (Figure S13a, Supporting Information). We added a pneumatic tube to each soft shell and sealed their inner volume (Figure S13b, Supporting Information). The inner pressure can both extend and retract the shell switching, therefore, between the two equilibrium points. We inserted 3D‐printed, stiffening rings in the shells’ lower and upper cylindrical sections to ensure proper retraction when depression is applied (without those rings, the shell with the original design shown in Figure S2d (Supporting Information) would collapse upon itself when exposed to depression since it has a completely soft structure). The pressure in the soft shells is controlled via 3‐port, 2‐position pneumatic valves that connect the shell volume to a low‐ or high‐pressure air supply (Figure S13c, Supporting Information depicts the corresponding pneumatic schematic). Moderate pressure values within −40 and +5 kPa are sufficient due to the low‐power requirements of this computational technique. Video S3 (Supporting Information) reports all the possible logic combinations performed with the pneumatically‐driven device.

**Figure 5:**
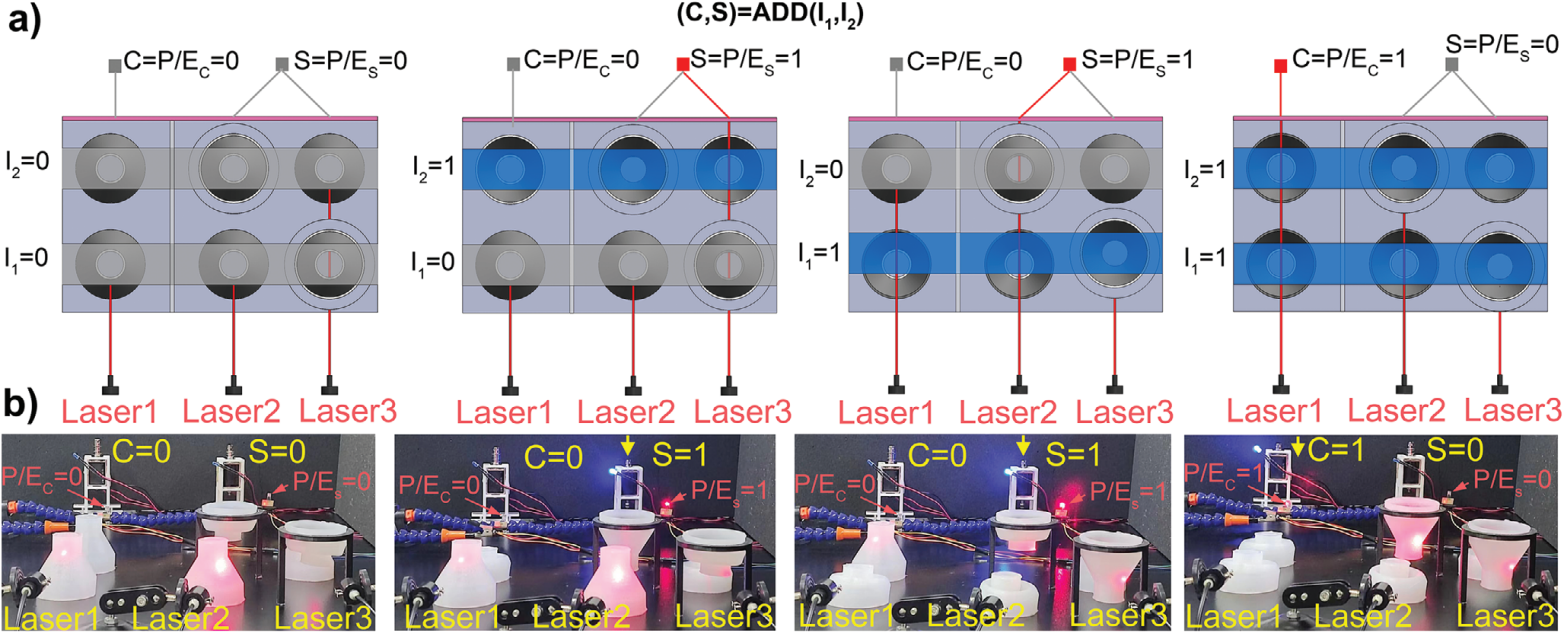
Experimental validation of the proposed half adder characterized by minimum implementation complexity when using an automated optical system (it relies on three laser emitters and one photoelectric converter, where the laser beams are blocked/transmitted depending on the soft shells’ state). a) Schematic of the four possible half adder configurations. b) Experimental photographs of the corresponding four scenarios when enabling/disabling LED lights and linear actuators for better visualization of the outputs (if an LED light is off and the corresponding actuator rod is lifted, then the output state is 0; conversely, the LED light becomes blue and the actuator rod is lowered when the output state is 1).

Finally, we have experimentally validated a complex logic element, namely the **half adder** depicted in **Figure** **5**a, to prove that the proposed approach is flexible enough to surpass simple logic gates. We assembled it with minimum complexity using an automated optical system. Laser beams can hit photoelectric converters (P/Es) that transform the optical signals into electric ones. These signals turn on/off two LED lights and operate two sliding actuators for a clearer visualization of the process (an external power supply provides the required energy). We assign the output values *C*  = P/E_C_  =  1 and *S*  = P/E_S_  =  1 when the laser beams hit the corresponding P/E so that the connected LEDs turn on and the actuator rod goes down (refer to the blue light and yellow arrows in the experimental photographs of Figure 5b). This initial result proves that our bistable soft shells are readily applicable to perform complex mechanical logic. Therefore, it is worth dedicating future efforts to incorporating these complex gates into a programmable device.

## 3 Conclusion

In summary, we have proposed a bistable, soft shell design with interchangeable internal/external surfaces, which is suitable for logic computation by allowing or stopping the transmission of laser beams for stable signal propagation. Using such a soft shell, we have created a buffer unit and a NOT gate crucial for developing six additional basic logic gates (namely the AND, NAND, OR, NOR, XOR, and XNOR gates). This list encompasses all the essential logic gates used to implement Boolean functions in computing systems so that our approach to performing mechanical computation is flexible and complete. Then, we have closed a gap in the literature by developing a rule‐changeable computing system tested with different actuation methods of the soft shells. This device includes all the basic logic gates mentioned above, ensuring comprehensive programmability options in situ (some soft shells are regarded as “setting units” to establish the desired logic gate, and others as “operational units” to perform computations). This technique eliminates the need for an independent design of each logic gate and broadens the device's suitability for multiple and continuous operations in a simple way, which is a key requirement often missing in the literature. Our systems can also be implemented with different sizes, even if we have targeted table‐scale dimensions. Upon proper modifications, it can enhance its computation capabilities, enabling more complex logic functions that go beyond the cited logic gates (for example, we have tested a half adder and addressed a full adder). Thus, our concept's mechanical nature conveniently supplements conventional electronic computation by offering unique advantages, such as working under extreme conditions and conveniently interacting with the environment. Future developments should apply physical embodiments of this principle to further elaborate electronic‐free, programmable devices that embrace more complex logic functions.

## 4 Experimental Section

### Soft Shells Prototyping

STL files of several molds were created in SolidWorks (version 2016) for prototyping the soft shells. These files were imported into an additive manufacturing system FS PFORMICS working with the laser‐powder bed fusion method (laser power 60 W, spot diameter 70 µm, scan speed 7.6 m s^−1^, deposition thickness 0.1 mm, hatch spacing 0.25 mm, and sintering zone temperature of 167.5 °C). The mold base material was nylon powder (FS3300PA, powder packing density 0.48 g/cm^3^, and particle diameter 10–50 µm). The resulting mold was used to obtain the soft shells as follows. The type A and B liquid silica gels (Yuchen, Guangdong China) were mixed in a ratio of 1:1 and thoroughly stirred them for ≈3 min. This mixture were injected into the cavities of these molds to fill them completely. A silicone curing time of ≈4–5 h was waited until solidification was reached. Finally, the soft structures were obtained by disassembling the reusable molds.

### Quasi‐Static Mechanical Experiments

A tensile test of the silicone material was performed based on the ASTM standards (Figure S2a, Supporting Information) using a universal mechanical testing machine (maximum force ≤ 85 kN, CMT5105, Shenzhen, China) with a tensile speed of 10 mm min^−1^ to obtain the constitutive model of shell material for subsequent analyses. Special shells for the quasi‐static experiments resembling the shape of a truncated cone were fabricated. They present four holes on the top surface and an extra bottom surface for anchoring (Figure S2c, Supporting Information). To record negative stiffness, the soft shells were connected to upper and lower holders (red structures in Figure 2a), which are fixed to the universal mechanical testing machine. The soft shells were compressed by imposing a displacement of 50 mm (twice the initial shell's height), which turns the external surfaces into the internal ones at maximum displacement. Moreover, the actual version of the soft shells for performing mechanical logic was prototyped with the same “molding–demolding” method. Compared to a pure truncated cone, the final version presents additional upper and lower cylindrical sections for easier actuation (Figure S2d, Supporting Information).

### Finite Element Simulations

The deformation response of the shells was stimulated with finite element analyses using Abaqus/Explicit procedure. The boundary conditions are shown in Figure 2a. The upper surface moved downward for 50 mm in each test, while the bottom surface was fixed. The shells were modeled using linear brick elements with reduced integration (C3D8R). According to a convergency study of the mesh, the total number of cells chosen for each design was ≈14000. The general contact algorithm with a tangential friction factor of 0.1 was applied to the model. The soft material (silica gel) was modeled using the basic Yeoh hyperelastic model. The form of the Yeoh strain energy per unit of reference volume *U_Y_* is:

(2) $$U_{Y}=\sum_{i=1}^{3}C_{i0}{\left(I_{1}-3\right)}^{i}$$

where $I_{1}$ is the first invariant of the right Cauchy–Green deformation tensor and *C*_*i*0_ refers to the material parameters. These parameters were obtained by fitting the strain–stress curve of the shell material shown in Figure S2a (Supporting Information) using Abaqus. They resulted equal to *C*_10_ =  3.86 · 10^−2^ MPa, *C*_20_ =  3.28 · 10^−3^ MPa, and *C*_30_ =   −6.24 · 10^−5^ MPa.

## Conflict of Interest

The authors declare no conflict of interest.

## Author Contributions

N.Y. performed conceptualization, methodology, supervision, and wrote the original draft and revision. Y.L., X.S., Z.M., and K.H. performed experiments. M.Z. performed simulation. D.P. performed methodology, supervision, wrote the original draft and revision.

## Supporting information



Supporting Information

Supplemental Video 1

Supplemental Video 2

Supplemental Video 3

## Data Availability

The data that support the findings of this study are available from the corresponding author upon reasonable request.
