# Supplementary material for: Bistable Soft Shells for Programmable Mechanical Logic
Source: Adv Sci (Weinh). 2024 Dec 6;12(5):2412372. doi: 10.1002/advs.202412372 (PMC11791939; doi:10.1002/advs.202412372)
Supplement: Supplementary file 1 — Supporting Information [file ADVS-12-2412372-s002.docx]

**Supporting Materials (SM)**

**Bistable soft shells for programmable mechanical logic**

*Nan Yang*^1^, Yuming Lan^1^, Miao Zhao^2^, Xiaofei Shi^1^, Kunpeng Huang^1^, Zhongfa Mao^1^, Damiano Padovani*^3^*

1. Intelligent Manufacturing Key Laboratory of the Ministry of Education, College of Engineering, Shantou University, Shantou 515063, China.

2. School of Mechanical and Electrical Engineering, University of Electronic Science and Technology of China, Sichuan 611731, China

3. Department of Mechanical Engineering (Robotics), Guangdong Technion-Israel Institute of Technology, Shantou 515063, China.

Corresponding authors: N.Y. ([nyang@stu.edu.cn](mailto:nyang@stu.edu.cn)) and D.P. ([damiano.padovani@gtiit.edu.cn](mailto:damiano.padovani@gtiit.edu.cn))

**Analytical model of the soft shells**

The elastic potential energy of a spring is defined as $U=\frac{1}{2}kx^{2}$, where $k$ is the spring stiffness (it can be linear or torsional stiffness) and $x$ is the spring deformation (it can be linear or angular displacement). We provide in Fig. 1b a simplified representation of the shell that is suitable for an analytical model of its potential energy by accounting for the changes of the equivalent linear springs’ lengths and torsion springs’ angles as follows:

| $U=k_{s}\left( l-l_{0} \right)^{2}+2k_{t}\left( \theta-\theta_{0} \right)^{2}=k_{s}\left( \sqrt{\left( h_{0}-h \right)^{2}+{d_{0}}^{2}}-\sqrt{h_{0}^{2}+{d_{0}}^{2}} \right)^{2}++2k_{t}\left[ \arccos\left( \frac{h_{0}-h}{\sqrt{\left( h_{0}-h \right)^{2}+{d_{0}}^{2}}} \right)-\arccos\left( \frac{h_{0}}{\sqrt{h_{0}^{2}+{d_{0}}^{2}}} \right) \right]^{2}.$ | (S1) |
| --- | --- |

Then, we have

| $\frac{U}{k_{s}}=\left( \sqrt{\left( h_{0}-h \right)^{2}+{d_{0}}^{2}}-\sqrt{h_{0}^{2}+{d_{0}}^{2}} \right)^{2}+2\frac{k_{t}}{k_{s}}\left[ \arccos\left( \frac{h_{0}-h}{\sqrt{\left( h_{0}-h \right)^{2}+{d_{0}}^{2}}} \right)-\arccos\left( \frac{h_{0}}{\sqrt{h_{0}^{2}+{d_{0}}^{2}}} \right) \right]^{2},$ | (S2) |
| --- | --- |

where $h$ and $h_{0}$ denote the compressive displacement and initial height of the shell, respectively, $k_{t}$ and $k_{s}$ are the stiffness of the torsion and linear spring, respectively, $d_{0}$ is the radius difference between the shell’s top and bottom parts marked in Fig. 1b, $l-l_{0}$ denotes the length difference between the actual elongation of the linear spring and its original state, and $\theta-\theta_{0}$ symbolizes the angle difference between the actual displacement of the torsion spring and its original state.

**Fabrication of the soft shells**

Figure S1 shows the different shapes and dimensions considered in this study to fabricate the lateral surfaces of the soft shells. Figure S2 illustrates the testing method used to assess the mechanical characteristics of the soft shells’ material and the procedures of the fabrication process.

| 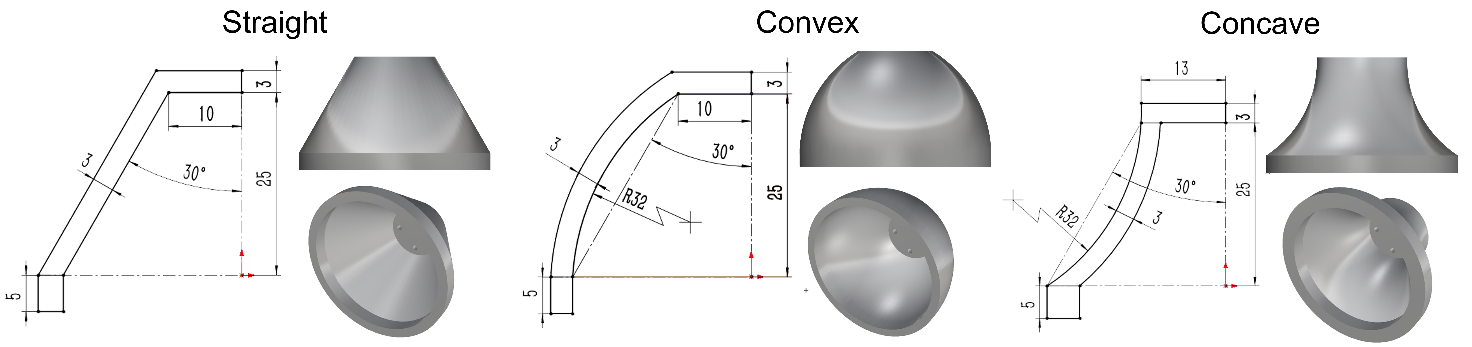  **Fig. S1**. Overview of the straight, convex, and concave shell designs with dimensions given in millimeters unless otherwise indicated. The shell nomenclature refers to the shape of its lateral surface. |
| --- |
| 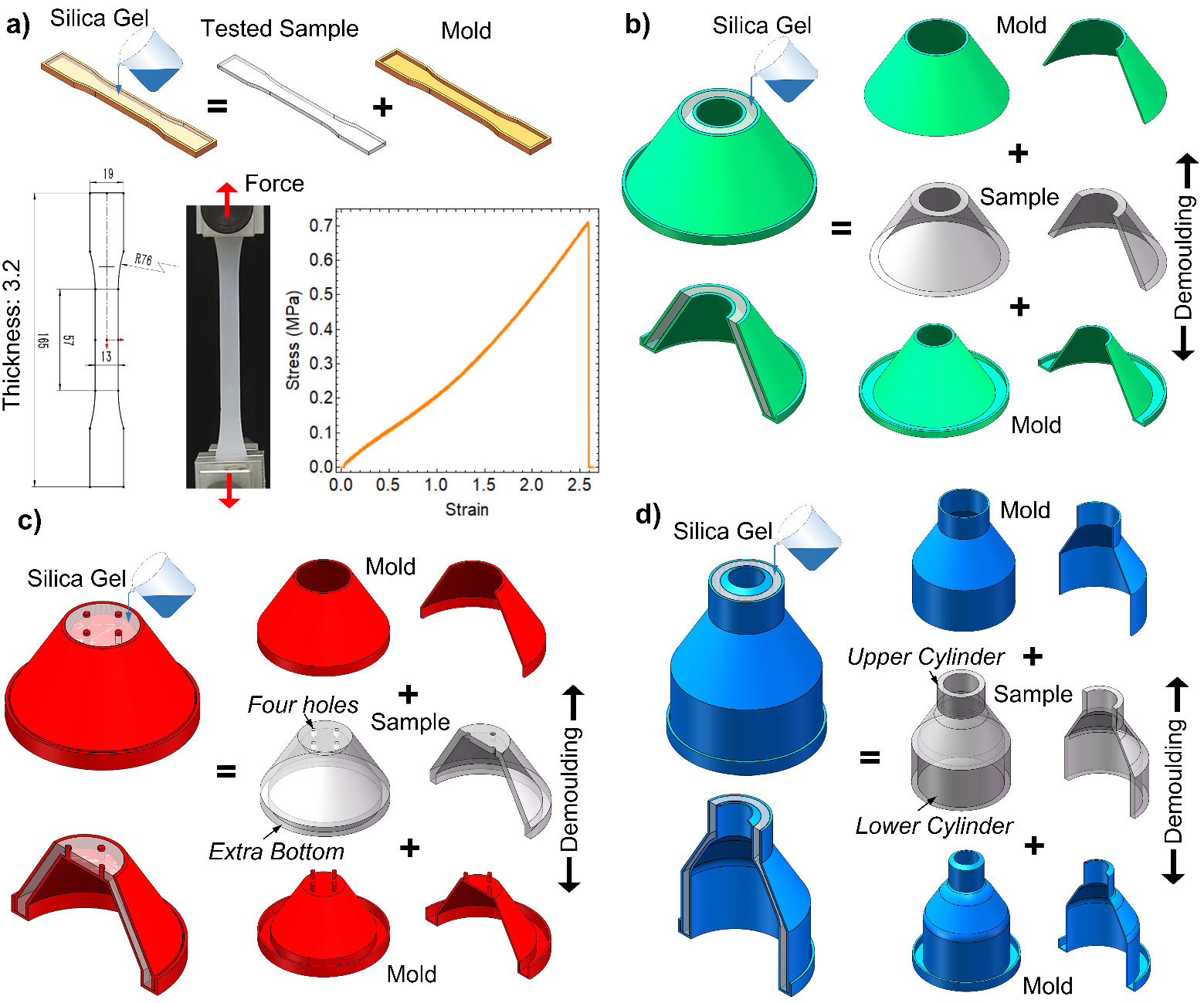  **Fig. S2**. Fabrication of the soft structures. (a) Fabrication of an ASTM type-1 tensile bar used to characterize the soft material’s mechanical properties (dimensions in millimeters, overview of the experimental setup, and stress-strain curve), (b) Fabrication process of soft shells with a pure cone profile, (c) Fabrication process of soft shells for the mechanical experiments reported in Fig. 2a, (d) Fabrication process of soft shells for actual computing operations (two cylindrical sections with reduced thickness are added below and above the conical section for easier actuation of the shells). |

We addressed the shell behavior regarding the compressive force needed to achieve variable displacements. Figure S3 refers to only one compression cycle involving different shell designs. Figure S4, on the contrary, presents the results of 100 compression cycles tested in a row for a shell with a straight profile and $\theta_{c}=30^{\circ}$, where the proposed shell’s manufacturing method guarantees a repeatable behavior over time.

| 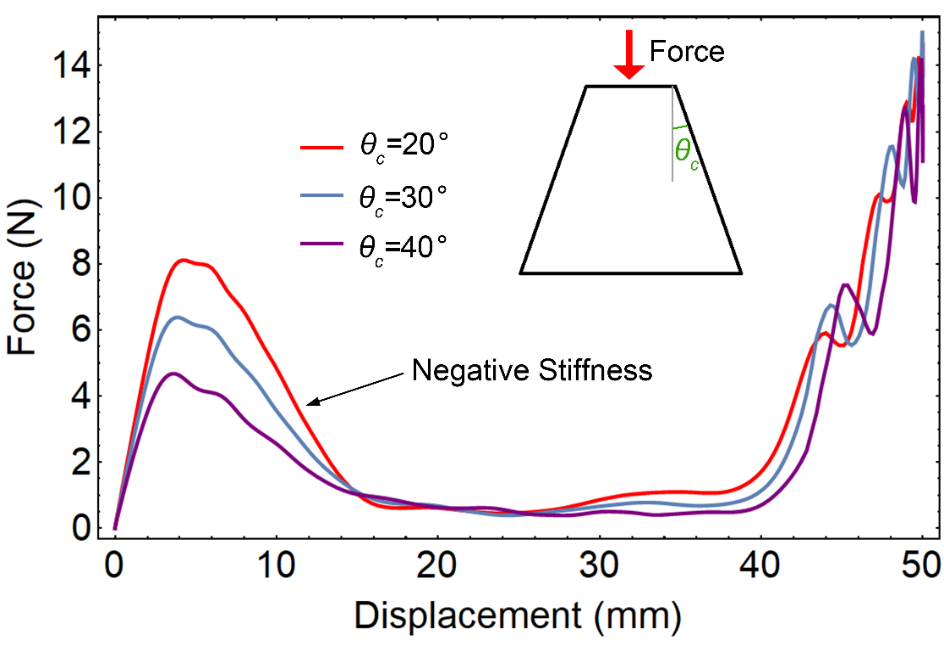  **Fig. S3.** Simulated force-displacement curves of straight soft shells with cone angles $\theta_{c}=20^{\circ}$, $30^{\circ}$, and $40^{\circ}$ and fixed cone’s top radius of 13 mm. A small value of $\theta_{c}$ requires larger compressive forces (we chose $\theta_{c}=30^{\circ}$ for our final implementation). All the investigated cone angles lead to negative stiffness, which results in the shell bistability. |
| --- |

| 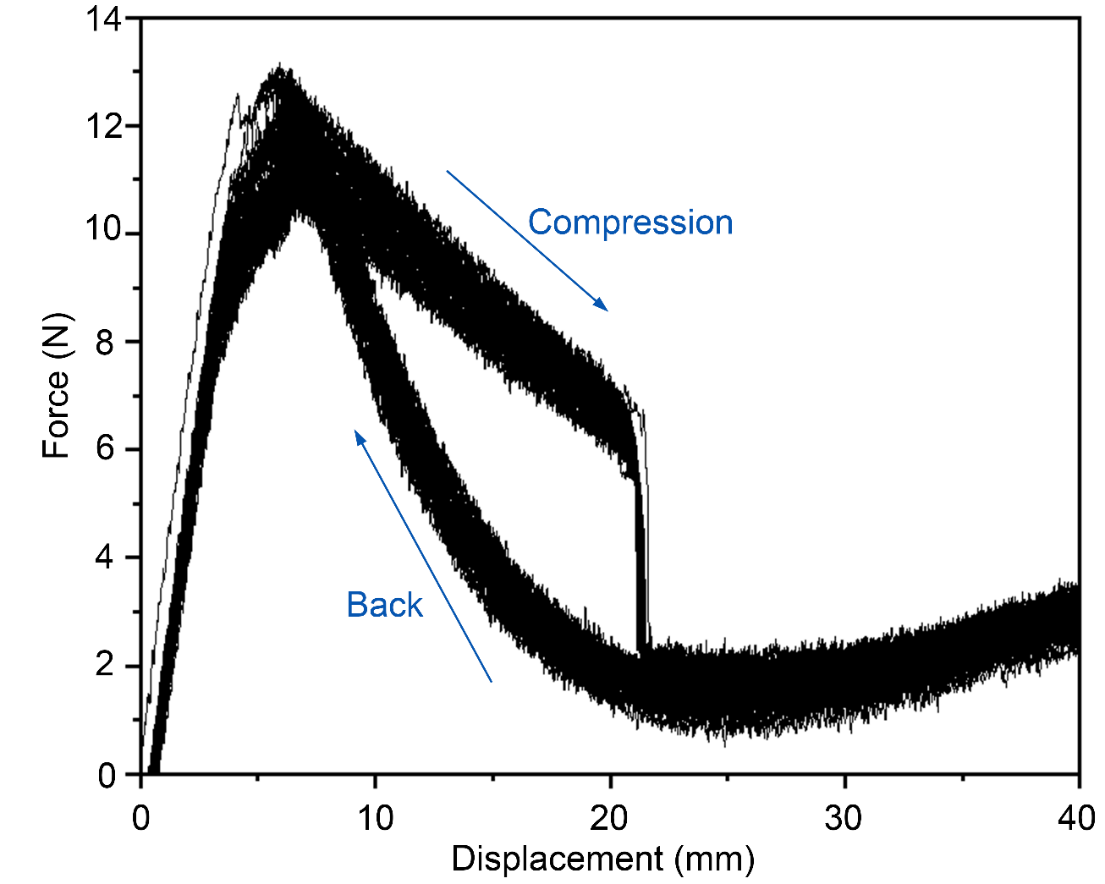  **Fig. S4.** Compression test of a straight soft shell repeated multiple times (100 cycles at a steady-state velocity of 40 mm/min). |
| --- |

**Implementation of the fundamental logic gates**

Figures S5, S6, and S7 depict a simplified implementation of the fundamental logic gates proposed in this study. These figures are meant to visualize each logic gate's operation principle clearly.

| 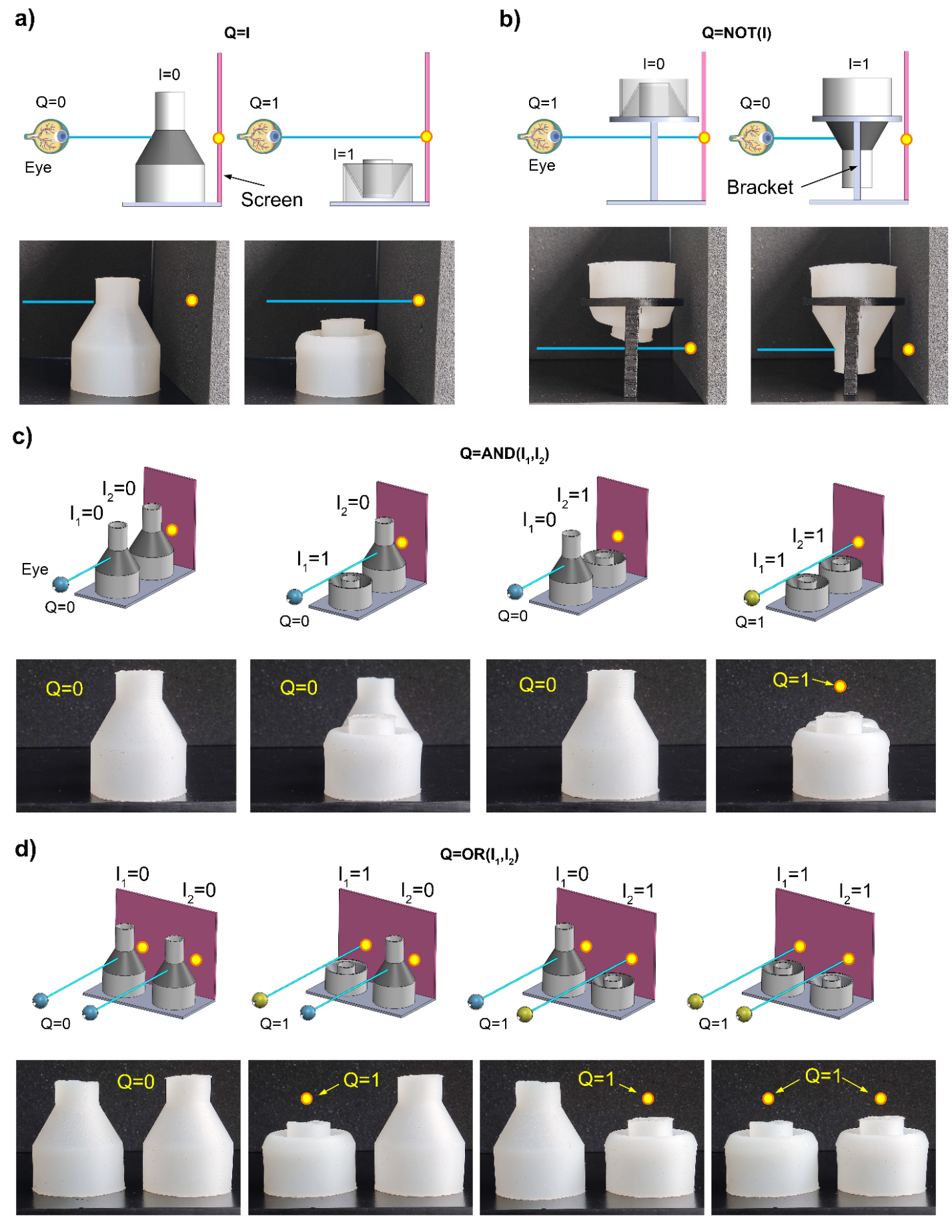  **Fig. S5**. Configurations of the proposed fundamental logic gates in all their states (**Part I**). (a) Buffer gate, (b) NOT gate, (c) AND gate, and (d) OR gate. The top row of each panel presents the system diagrams that highlight the inputs and outputs of the gate, the interrupted/uninterrupted lines of sight (segments in blue), and the targets on the screen (yellow dots). The bottom row depicts experimental photographs along with the indication of the targets. |
| --- |
|  |
| 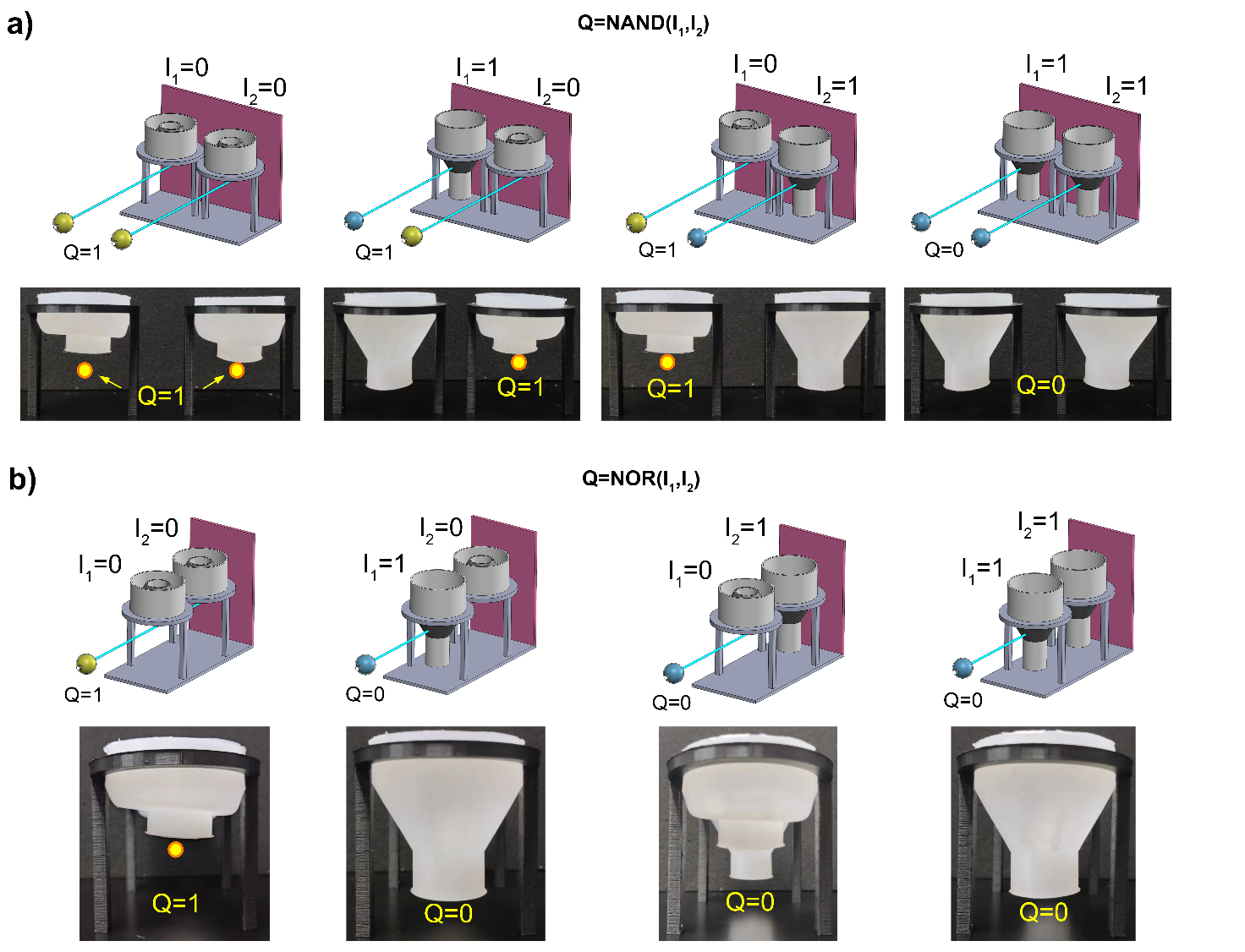  **Fig. S6**. Configurations of the proposed fundamental logic gates in all their states (**Part II**). (a) NAND gate and (b) NOR gate. The top row of each panel presents the system diagrams that highlight the inputs and outputs of the gate, the interrupted/uninterrupted lines of sight (segments in blue), and the targets on the screen (yellow dots). The bottom row depicts experimental photographs along with the indication of the targets. |

| 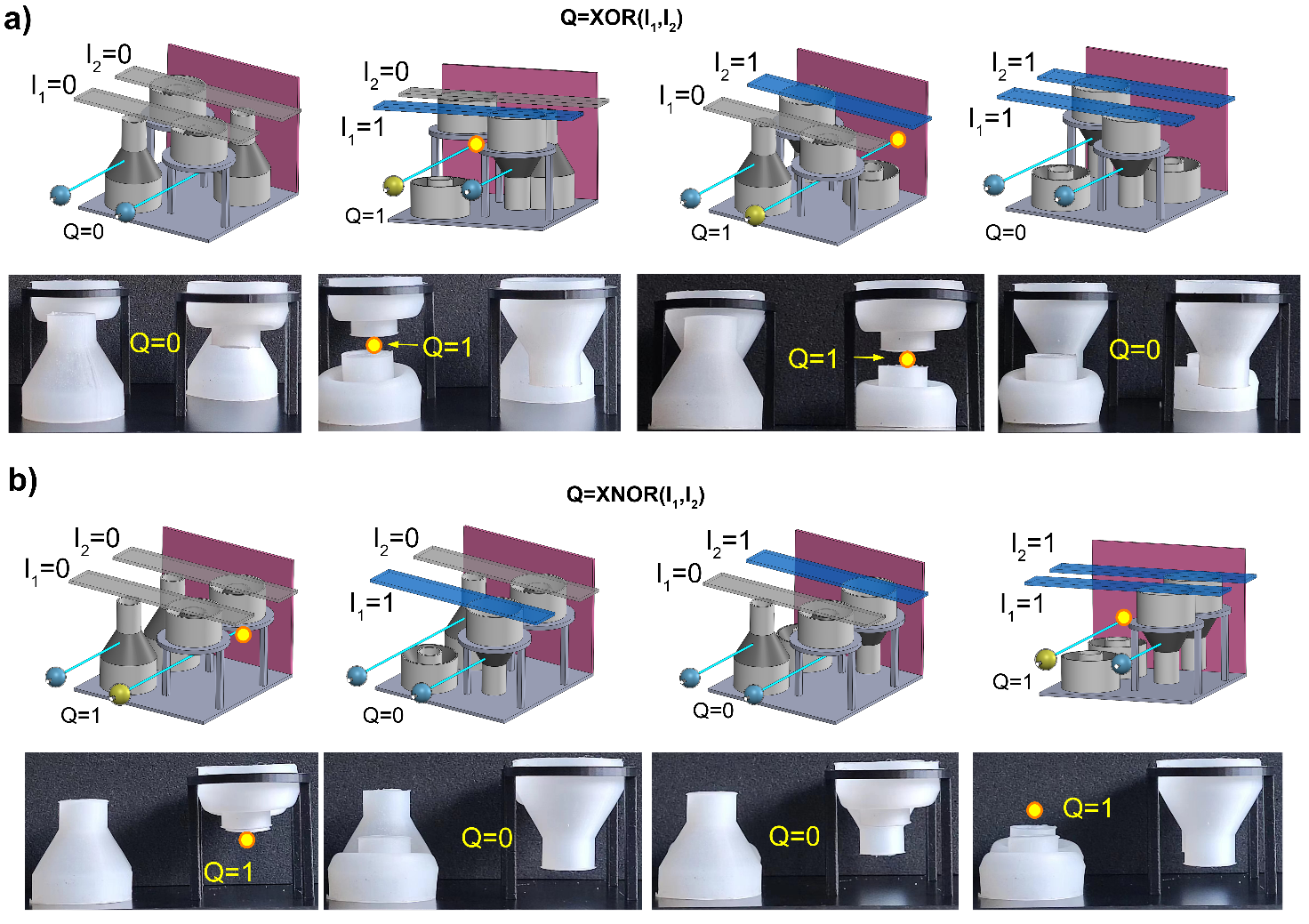  **Fig. S7**. Configurations of the proposed fundamental logic gates in all their states (**Part III**). (a) XOR gate and (b) XNOR gate. The top row of each panel presents the system diagrams that highlight the inputs and outputs of the gate (the inputs are indicated by strips that trigger two basic units simultaneously), the interrupted/uninterrupted lines of sight (segments in blue), and the targets on the screen (yellow dots). The bottom row depicts experimental photographs along with the indication of the targets. |
| --- |

**Implementation of complex logic gates**

Moving toward more complex operations, we can arrange our fundamental logic elements to build digital circuits that perform the addition of numbers. We initially propose a **half adder** by joining an AND gate with an XOR gate. This device adds two single binary digits (the inputs $I_{1}$ and $I_{2}$) to deliver two outputs in the form $\left( C,S \right)=\mathrm{ADD}_{Half}(I_{1},I_{2})$, namely the sum $S=I_{1}\oplus I_{2}$, where the operator $\oplus$ outputs true only when the inputs differ, and the carry $C=\mathrm{AND}(I_{1},I_{2})=I_{1}\cdot I_{2}$. This combination of an AND gate for the carry and an XOR gate for the sum is separated by an isolation panel for clarity in Fig. S8a. Then, the three soft units below each transparent strip are simultaneously subjected to the same input. This arrangement gives four output scenarios, namely $\left( C,S \right)=\left( 0,0 \right)$ if no units are triggered, $\left( C,S \right)=\left( 0,1 \right)$repeated twice when only $I_{1}$ or $I_{2}$ is active, and $\left( C,S \right)=\left( 1,0 \right)$ if both $I_{1}$ and $I_{2}$ are triggered.

Additionally, we introduce a **full adder** by leveraging our fundamental elements (buffers and NOT gates), as depicted in Fig. S8b. This device is represented in the form $\left( C,S \right)=\mathrm{ADD}_{Full}(I_{1},I_{2},I_{3})$ because it adds three single binary numbers (the inputs $I_{1}$, $I_{2}$, and $I_{3}$) to deliver two outputs, precisely the sum $S=I_{1}\oplus I_{2}\oplus I_{3}$ and the carry $C=I_{1}\cdot I_{2}+I_{2}\cdot I_{3}+I_{3}\cdot I_{1}$. At this stage, we replace the device operator with an automated optical mechanism due to the increased complexity of the logic (in general, the outputs $\left( C,S \right)$ can be electrical, optical, or mechanical signals based on the need). Implementing this rule requires seven laser sources, two photoelectric sensors, and eighteen soft shells, where each group (three groups of six shells in total) underneath each transparent strip in the upper portion of Fig. S8b is triggered/untriggered at the same time. This arrangement leads to eight working conditions reported in the lower section of Fig. S8b, namely $\left( C,S \right)=\left( 0,0 \right)$ when no shells are triggered (all inputs are $I_{i=1,2,3}=0$), $\left( C,S \right)=\left( 1,1 \right)$if all units are triggered (all inputs are $I_{i=1,2,3}=1$), and $\left( C,S \right)=\left( 0,1 \right)$or $\left( 1,0 \right)$ recurring three times each when at least one input differs from the remaining two.

| 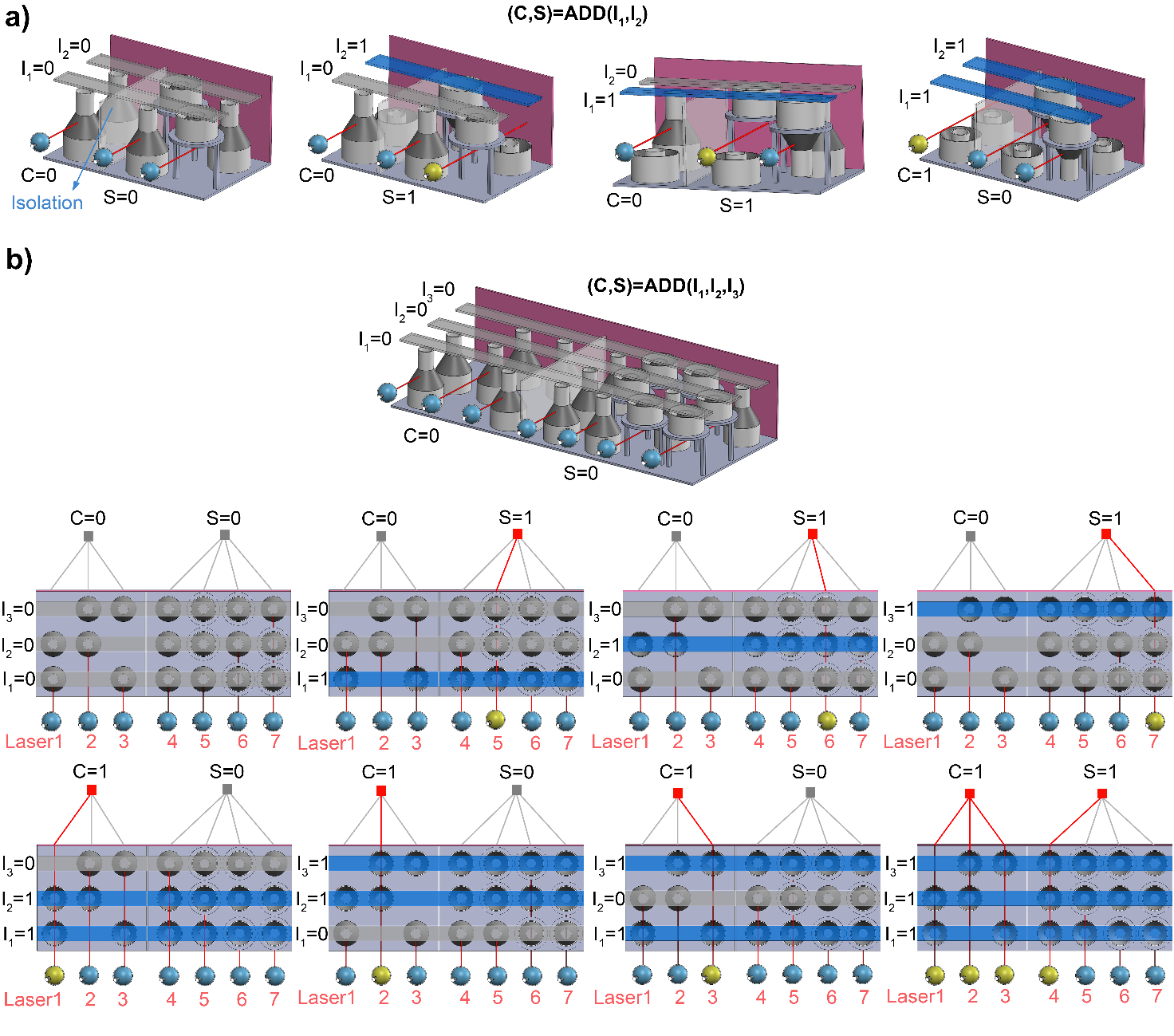  **Fig. S8**. System diagrams (overviews and top views) of the proposed complex logic elements based on multiple buffers and NOT gates: (a) Half adder with its four possible configurations and (b) Full adder with its eight possible configurations. These implementations involve laser beams and photoelectric converters to handle the device outputs (the carry $C$ and the sum $S$), while the mechanical inputs ($I_{i=1,2,3}$) are indicated by equivalent strips that trigger several soft units simultaneously (the blue color of those strips refers to the input being triggered). |
| --- |

**Implementation of programmable (fundamental) logic gates**

Figure S9 reports a comprehensive overview of the achievable configurations *in situ* with the rule-changeable device developed in this research, while Fig. S10 illustrates the experimental validation of the same scenarios.

| 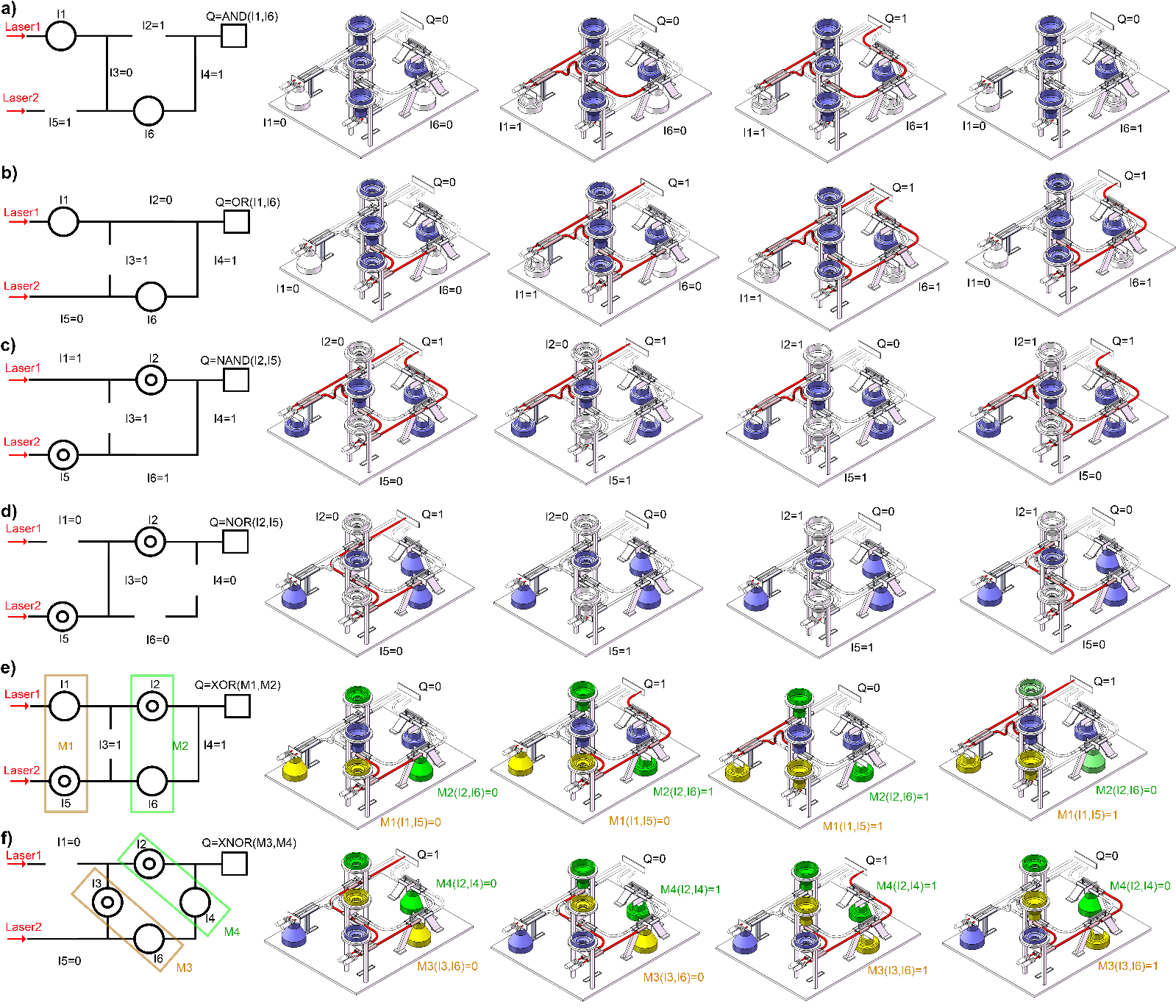  **Fig. S9**. Analysis of the rule-changeable device that performs multiple fundamental mechanical logic gates according to all their possible configurations (from top to bottom: (a) AND gate, (b) OR gate, (c) NAND gate, (d) NOR gate, (e) XOR gate, and (f) XNOR gate). The shells highlighted in blue are those units used to enable each logic gate (“setting units”), while the remaining shells are those used to apply the input signals (“operational units”). The paths of the laser beams operated in each scenario are highlighted in red color. For the XOR gate and XNOR gate, the units encapsulated within each frame in the equivalent diagram on the left use the same input and are activated simultaneously (their behavior is described as $M_{i=1,2,3,4}\left( a,b \right)=c$, where $a=b=c$, *e.g.*, in (e) $M_{1}\left( I_{1},I_{5} \right)=1$indicates $I_{1}=1,I_{5}=1$). |
| --- |

| 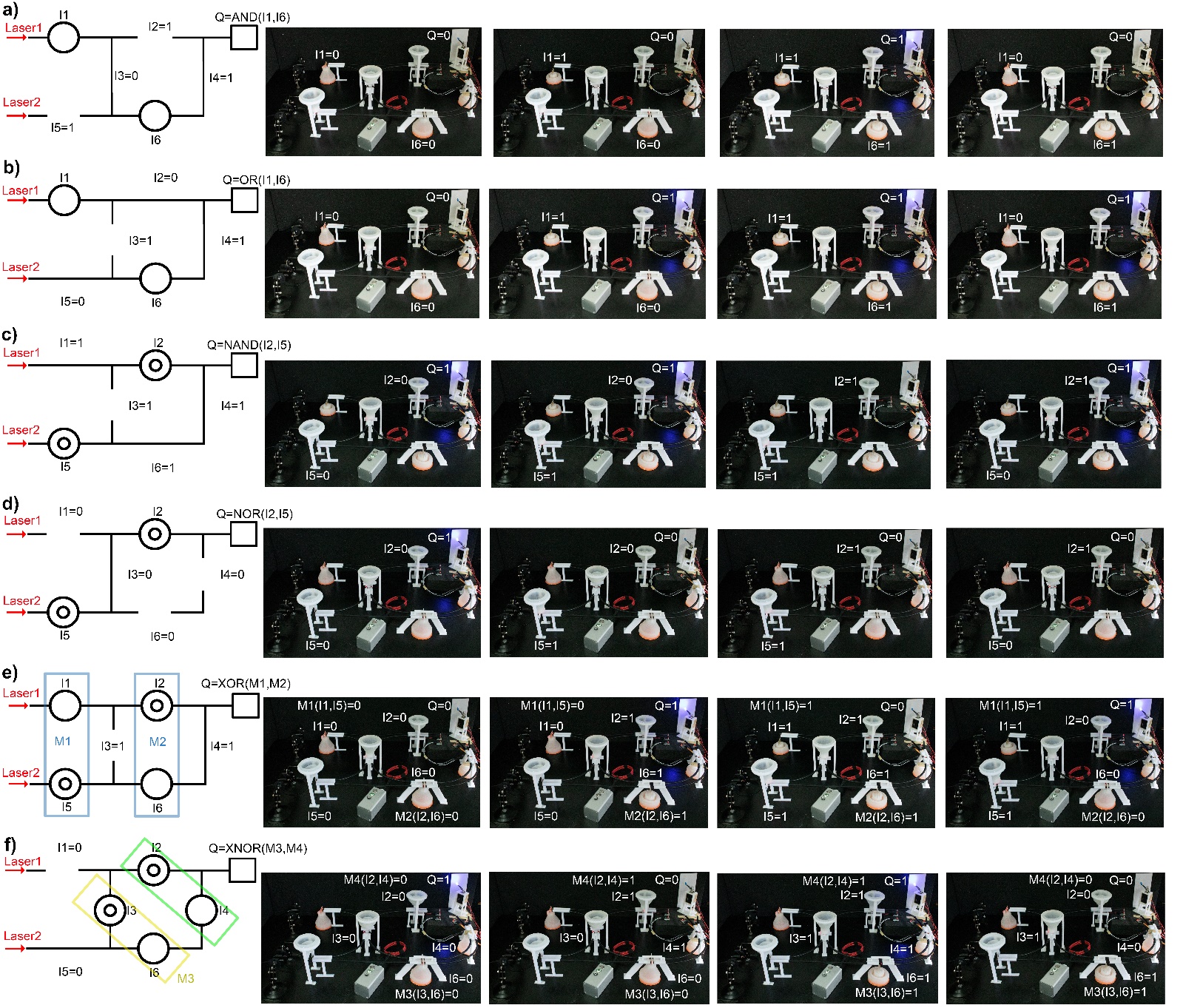  **Fig. S10**. Experimental photographs of the rule-changeable device that performs multiple fundamental mechanical logic gates according to all their possible configurations stated in Fig. S9 (from top to bottom: (a) AND gate, (b) OR gate, (c) NAND gate, (d) NOR gate, (e) XOR gate, and (f) XNOR gate). |
| --- |

Figure S11 shows the mechanism for visualizing the output signals of the programable device using electromechanical relays.

| 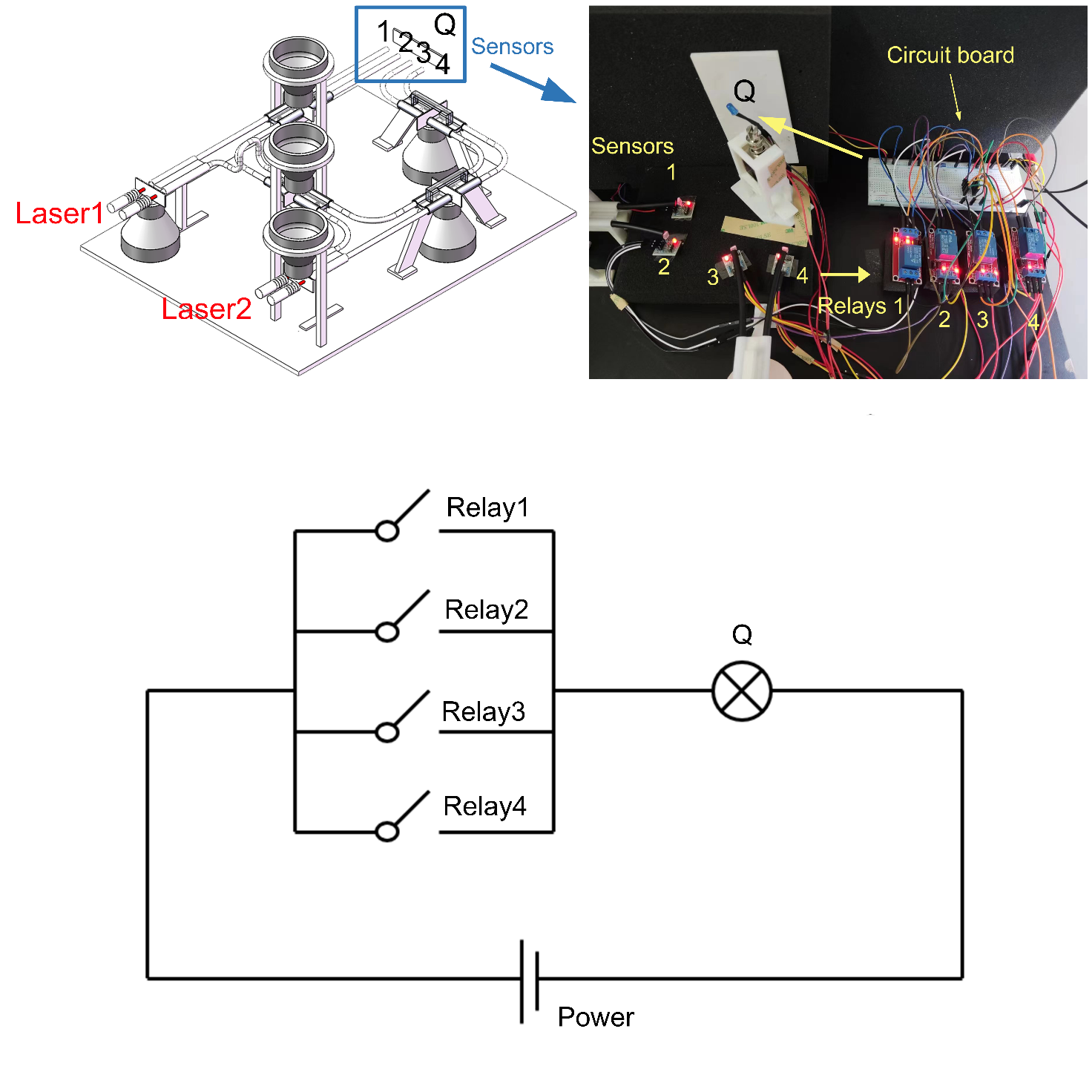  **Fig. S11**. Generation of the output $Q$ in the experiments reported in Fig. S10. Top: overview and labeling of the laser sensors 1, 2, 3, and 4 located at the optical fibers’ end and of the corresponding relays 1, 2, 3, and 4 connected to the circuit board used to generate the values of $Q$ (*i.e.*, the status of the LED light and of the linear actuator). As a result, the output becomes $Q=1$ when, at least, one sensor receives the laser beam. Bottom: simplified electric diagram of the circuit used for visualizing the output $Q$ (each laser sensor commands its corresponding relay). |
| --- |

Figure S12 gives an example of the opportunity to realize combinational logic by expanding the basic programmable device shown in Fig. 4a.

| 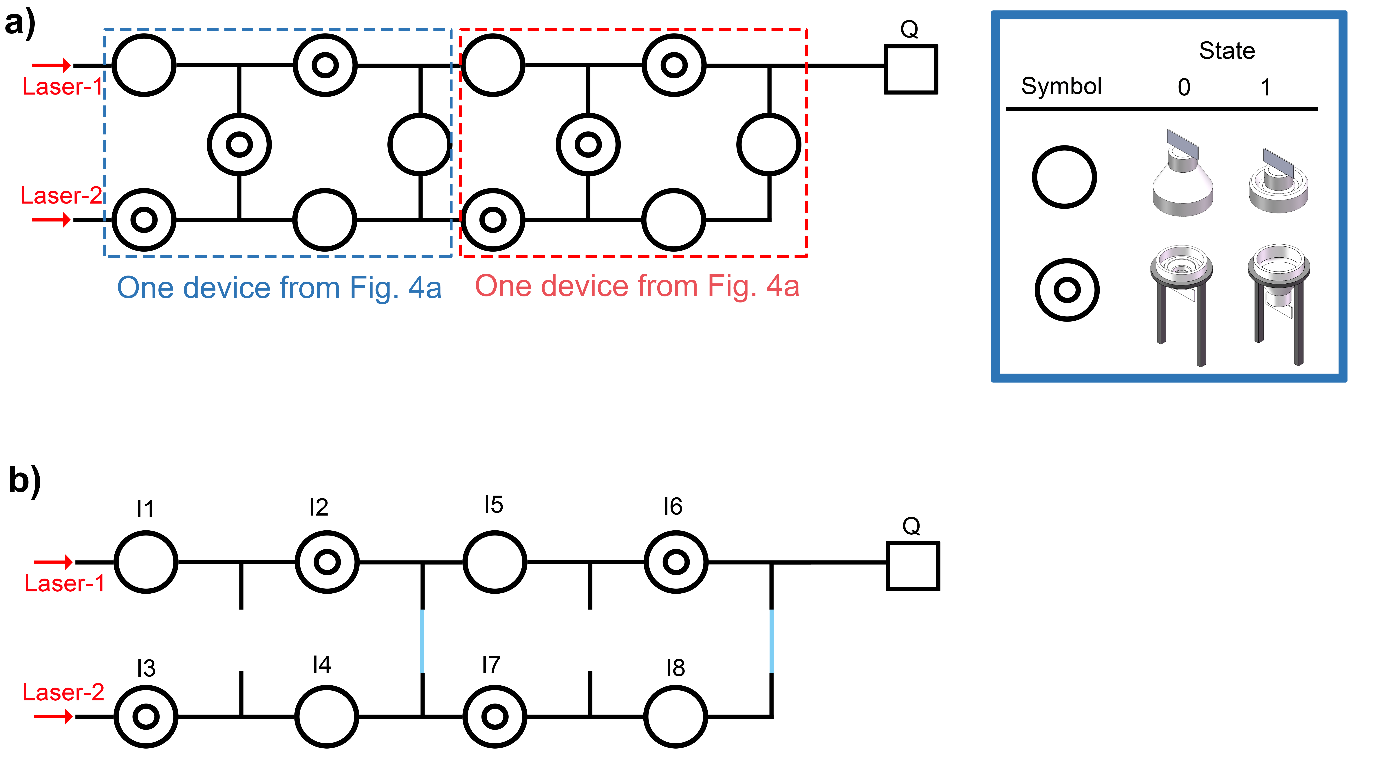  **Fig. S12**. (a) A conceptual representation of a rule-changeable system with an extended design combining basic programmable devices shown in Fig. 4. (b) This system can realize combinational logics for enhanced functions, as an example the scenario $Q=(I_{1}\cap\bar{I_{2}}\cup I_{4}\cap\bar{I_{3}})\cap(I_{5}\cap\bar{I_{6}}\cup I_{8}\cap\bar{I_{7}})$, where $\cap$ and $\cup$ denote AND and OR rules, respectively. |
| --- |

Lastly, Fig. S13 refers to the programmable device operated with air-driven actuation, which is a pneumatic version of the one in Fig. 4. The pneumatic components are directional control valves 3H210-08 (they are bistable devices operated manually) and tubes with 6-millimeter diameter. A compressor (Eluan E8L-550W) supplies the high-pressure line controlled by a pressure regulator (ZKAY IR2000-02BG) with a setting equal to 5 kPa (this pressure value was verified with an electronic pressure transducer Festo SPTW-P2R-G14-A-M12). The low-pressure line contains a 24-liter air reservoir pressurized at –20 kPa with a vacuum pump (VP215 Series HVAC) before running the experiments (a ball valve closes the connection to the vacuum pump); due to the reservoir’s large volume, this pressure remains about constant.

| 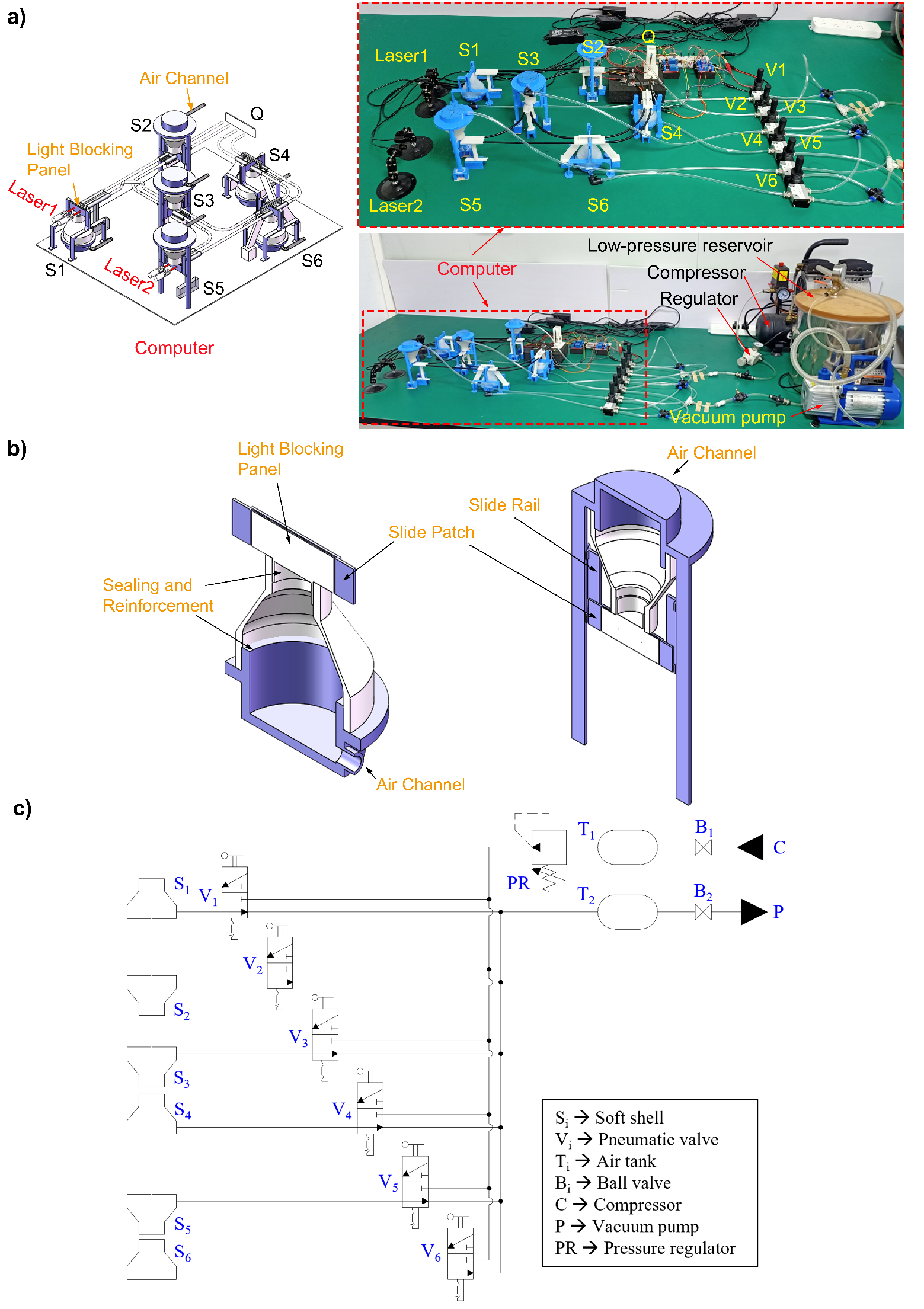  **Fig. S13**. (a) Overview of the programmable device introduced in Fig. 4a when modified to become air-driven. (b) Detailed view of the reinforced and sealed soft shells suitable for pneumatic operations (sliding patches and rails are used for unidirectional motion of the light-blocking panels). (c) Pneumatic diagram of the system used to drive the programmable device for mechanical computations. |
| --- |

**Supporting videos**

We provide three videos to visualize our experimental validations:

- **Video 1** refers to the programable device shown in Fig. 4a, which is hand-driven in this case (the inputs of the soft shells are assigned manually by the operator). This video covers testing the six fundamental logic gates (AND, OR, NAND, NOR, XOR, and XNOR) in all configurations.
- **Video 2** demonstrates the expected functioning of the programable device shown in Fig. 4a when a strong electromagnetic disturbance is applied nearby the air-driven soft shells (we only report all the configurations of the AND gate for brevity).
- **Video 3** reports testing the same scenarios included in Video 1 (all the configurations of the six fundamental logic gates). The programable device introduced in Fig. 4a is now air-driven (pressure signals assign the inputs of the soft shells), as shown in Fig. S13.

**Highlights:**

1. We propose a general method for forming electronic-free computers based on all signal transmission means.
2. We develop a rule-changeable embodiment to easily perform all the essential gates for Boolean functions *in situ*.
3. We test manual and automatic methods for operating the proposed computing system.
4. We prove that such a system can smoothly work under harsh conditions, such as electromagnetic perturbations.
